# Water intake: validity of population assessment and recommendations

**DOI:** 10.1007/s00394-015-0944-8

**Published:** 2015-06-06

**Authors:** Joan Gandy

**Affiliations:** School of Life and Medical Services, University of Hertfordshire, Hatfield, AL10 9AB UK

**Keywords:** Water intake, Dietary assessment methodologies, Hydration, Fluid intake, Recommendations

## Abstract

Good hydration is vital for good health and well-being. Until recently, there was little interest in collecting data on water and drink and beverage intake. However, there is increasing evidence that a low water intake or mild dehydration may be linked with the risk of chronic diseases. Accurate estimates of intake in populations are essential to explore these relationships. This will enable the identification of specific populations at the risk of low water intake and allow exposure assessment of potential contaminates and specific nutrients present in drinks and beverages. In addition, data from these population studies are used as the basis of national and international recommendations on water intake and to set and evaluate national health policies. For example, EFSA based their recommendations on data from population studies from 13 European countries. The range of intakes varied from 720 to 2621 mL/day; this diversity cannot be explained by environmental differences alone. However, this variability may, at least partially, be explained by the inconsistency in methodologies used as none of surveys used a dietary assessment tool validated for total water intake or beverage and drink intake. It is reasonable to suggest that this may result in incomplete data collection and it raises questions on the validity of the recommendations. The relationship between water consumption and health warrants further investigation, and robust methodologies are essential to ensure that these data are accurate and useful for setting public health priorities and policies.

## Introduction

There is increasing evidence of the links between water intake and physical disease and cognitive performance [1–3]. The prevalence of dehydration in adults has been estimated to be 16–28 % depending on age [4] with the elderly being at increased risk of dehydration and associated morbidities [5, 6]. However, to establish health and disease risk relationships in all age groups with intake and to make reliable recommendations on water intake, it is essential to have accurate estimates of intake in populations. Current methodologies need to be improved to ensure accurate data before such relationships can be fully explored. This will inform both recommendations and public health programs.

## Recommendations on total water intake

The sources of water are fluids, or beverages, (including drinking water and water in fluids, e.g., tea, wine, soft drinks) and water in food. There is often confusion about the use of the term “beverages” or “fluids” to represent water intake apart from water in food; for the purposes of this review, the term drinks and beverages will be used. All foods contain water although the amount of water in a food will vary between individual foods and diets. The European Food Safety Authority (EFSA) estimated that 20–30 % of total daily water intake in Europe comes from food [7]. However, the overall percentage of water in foods will vary between countries and seasons and depend on food types and dietary patterns. For example, in Ireland, it is estimated to be 33 % [8] and in 40 % in four cities in China [9] where more liquid foods, e.g., soups, broths are consumed.

Both EFSA [7] and the Institute of Medicine (IOM) [10] in the USA have published gender- and age-specific recommendations on water intake as shown in Table 1. However, they took slightly different approaches in determining their recommendations. The IOM concluded that it was not possible to give an estimated average requirement due to extreme variability in water requirements that cannot be explained by different metabolism, variability in environmental conditions and activity. Moreover, the scientific evidence available at that time was insufficient to establish an intake level of total water intake that would reduce the risk of chronic diseases. Therefore, IOM set adequate intakes (AI) based on median intakes observed in national surveys, which form the basis of recommended daily allowances [10]. In contrast, EFSA [7] based their AIs on “a combination of observed intakes in population groups with desirable osmolarity values of urine and desirable water volumes per energy unit consumed.” They also recommended that the AIs only apply in moderate environmental temperatures and at moderate physical activity levels (PAL 1.6).

**Table 1:** Dietary reference intakes (adequate intakes) for total water set by the European Food Safety Authority (EFSA) and the Institute of Medicine (IOM)

	Adequate intake (L/day)			
	EFSA [3]		IOM [24]	
Age	Total water intake	Fluid intake^a^	Total water intake	Fluid (beverage) intake)
0–6 months	0.68^b^	0.68^b^	0.70	0.70
6–12 months (IOM 7–12 months)	0.80–1.00	0.64–0.80	0.80	0.80
1–2 years	1.10–1.20	0.88–0.90		
2–3 years	1.30	1.00		
1–3 years			1.30	0.90
4–8 years	1.60	1.20	1.70	1.20
9–13 years				
Boys	2.10	1.60	2.40	1.80
Girls	1.90	1.50	2.10	1.60
>14 years as adults				
Boys	2.50	2.00	3.30	2.60
Girls	2.00	1.60	2.30	1.80
Adults				
Men	2.50	2.00	3.30	2.60
Women	2.00	1.60	2.30	1.80
Pregnant women	+0.30		+0.30	0.10
Lactation women	+0.60 to 0.70		+1.10	0.90
Elderly	As adults	As adults	As adults	As adults

^a^80 % of total water intake; ^b^ through milk

The population intake data used by IOM are a single data set using the same methodology within one, albeit climatically diverse. However, EFSA used population surveys from European countries with average intakes varying from 720 mL/day to over 2621 mL/day [7]. Countries of similar climate and cultural backgrounds showed very diverse total water intakes and patterns of the types of beverages consumed. This disparity throughout Europe cannot be entirely explained by differences in environmental temperature or culture suggesting that differing assessment methodologies may be responsible for some, if not most, of the variation.

While only the examples of EFSA and IOM guidelines have been discussed here, there are many examples of guidelines for individual countries or regional groups with variable recommendations [7]. One example of this is the daily drinks recommendations for the elderly men, which vary 1.0–1.5 L/day–3 L/day [5].

## Dietary assessment methodologies

The selection of a dietary assessment method depends on the research question and whether data for an individual or a population are needed. However, dietary assessment has inherent errors including under-reporting and, even given modern technology facilitating real-time tracking, no single method will give an entirely true assessment of intake of any particular nutrient [11]. In addition, there must always be a balance between the accuracy of an assessment method and the burden it imposes on the respondent. This is particularly true when surveying populations in which the aim of the study is to look for dose–response relationships [12].

All dietary assessment method has different limitations and advantages [13]. For example, retrospective methods, e.g., 24-h recall, diet history are dependent on respondents’ memory and recall abilities, and in the case of a diet history, it may be influenced by the present diet. Respondents must be able to use conceptualization skills to describe and estimate frequency of consumption and portion size [14]. Food frequency questionnaires (FFQ) are frequently used in population studies of diet. While cost-effective and requiring minimal effort from respondents they are retrospective; it is advisable that they be validated in the study population [15]. Prospective methods, e.g., weighed or estimated food diaries do not require memory or conceptualization skills and are completed in real time. However, they are more expensive and require greater cooperation from the respondents; in addition, the process of recording may influence intake.

The choice of a methodology will depend on many factors, but perhaps the most important is the ability of the methodology to capture a particular nutrient or nutrients in the population of interest [16]. The nutrients of interest are often decided by emerging health issues within the population. For example, with the increasing rates of obesity, there is much emphasis on capturing energy intakes as accurately as possible by the use of doubly labeled water [17]. In addition, dietary assessment methods must be validated by comparison with a reference method or biomarker. Methodologies are validated against what is believed to be the most accurate measure of that nutrient. For example, an instrument that assesses energy intake can be validated against doubly labeled water studies and protein can be validated against nitrogen balance. However, population surveys aim to capture many nutrients and should be validated accordingly. An example of this is the validation of tools used in the UK arm of the EPIC study [18]. Population surveys to date have concentrated on assessing macro- and micronutrient intakes but not water intake. Therefore, the methodologies employed have not been validated for the assessment of total water intake or beverage and drink intake.

However, this and other surveys were designed to study food and nutrient intakes; drinks and beverages, especially water, may not always have been recorded. In more recent surveys, where drinks and beverages have been recorded, this information is collected by the extension of the existing food record without due consideration of drinking behavior. Food intake is usually structured around meals, often three meals a day with occasional snacks [19]. However, beverages and drinks are drunk at meal times and throughout the day [20], often without consumption of energy, and may therefore not always be recorded. In addition, the choice of beverage or drink may vary across the day. For example, alcohol is more likely to be consumed in the evening [20]. Therefore, a diary structured around meals is likely to underestimate total water intake because they are designed primarily to capture foods and extensions of this technique are unlikely to be sensitive enough to capture all drinking events.

## Calculation of nutrient intake

To calculate nutrient intake from intake data collected from dietary assessment, foods are given codes, which relate to a specific food within a food composition table or database. Using the recorded, weighed or estimated portion size, nutrient intake is calculated. These databases and tables must be specific to the study population and have inherent limitations [21] including coding errors. In large studies, automated systems such as the USDA’s dietary intake data system [22] are used; therefore, it is essential that foods and drinks and beverages be coded correctly to minimize errors. However, until recently, not all tables and databases included a code for water. For example, in a study of preschool children, water was excluded, as it was not a part of the food and nutrient database used to calculate intakes [23]. This raises concerns that beverage and drink intakes may have been underestimated.

At present, there is also the issue that in order to establish total water intake it is necessary to calculate and then total the components of water intake separating, e.g., beverage and drink intake water and water in food. As discussed, water in food is not always available in food composition tables and metabolic water is rarely, if ever, calculated. These issues will add to the unreliability of total water intake data from population surveys.

## Methodologies to assess total water intake and beverage and drink intake

Unsurprisingly, the quality of data on water consumption in national studies has been questioned, as the use of inconsistent methodologies will probably result in incomplete data collections [1]. Sebastian et al. [24] compared drinking water intake in two cohorts of the What We Eat in America/National Health and Nutrition Examination Survey (NHANES) and found significant differences in water intake between the 2005–2006 survey that used the Automated Multiple-Pass Method for 24-h recall and the 2003–2004 survey that used post-recall food frequency-type questions.

The diversity in methodologies used by nine of the European countries by EFSA is shown in Table 2; information on the remaining countries was not available. The methodologies used included dietary recalls of varying length, FFQs and 7-day records or a combination of methods. While the wide range of intakes in Europe will be multifactorial given the diversity of methods used, it is reasonable to conclude that methodology is an important factor. Since the EFSA AIs are based on these intakes, the validity of the recommendations could therefore be questioned.

**Table 2:** Methodologies used in nine European countries’ population surveys included in data used by EFSA to establish adequate intakes of water [43]

Country	Methodology
Hungary	3 × 24 h recall
Norway	FFQ^a^
Iceland	24-h recall
Ireland	7-day record
Belgium	2 × 24 h recall + FFQ
UK	7-day record
Italy	7-day record
Germany	4-week recall + FFQ
France	7-day record

^a^ *FFQ* food frequency questionnaire

This focus on water intake is relatively new, and therefore, the science needed for robust methodologies is poorly developed [1]. As a consequence, there is a reliance on existing methodologies that have been validated for other nutrients. In a recent systematic review of fluid intake in beverages across all ages [25], the most frequently used method was 24-h recall (29 out of 65 studies) of which 22 were single 24-h recalls. Two studies used specific tools: a 7-day fluid diary [26] and a beverage dietary history [27]. A single 24-h recall is not considered representative of typical individual diet due to day-to-day variations and inherent limitations of the method reliance on memory, the need for conceptualization skills and under- or over-reporting [28]. While it is considered suitable for epidemiological studies, the specific population to be studied must be considered and appropriate validation conducted. For example, in a study conducted to select a dietary assessment method in a low-income population, four repeated multipass 24-h recalls were recommended [29].

As with all dietary assessment tools, an assessment method capable of capturing all drinking events should be validated against a criterion measure. The approaches may include using deuterium dilution as a measure of total hydration or by using as a measure of hydration such as plasma osmolality. An alternative is the direct observation of intake as used by Jimoh et al. [30]. They recently reported the development of a drinks diary for use in the elderly. In a pilot study, the self-reported drinks diary showed high correlation (Pearson’s correlation coefficient *r* = 0.93) with direct observations. Interestingly, this study also highlighted the inaccuracy and unreliability of care staff records.

While there is no consensus on how to assess water intake in health, there is a recommended methodology for water quality and exposure studies. An international panel reviewed the literature on the topic and concluded that the best method for collected water intake data was a 3- to 4-day diary. However, if this is not feasible, repeated 24-h recalls are preferable to a FFQ [31]. This suggests that there may be a benefit of crossdiscipline working to share experience and work toward a consensus on methodologies for collecting accurate data on water consumption in health studies.

Regardless of the methodology’s ability to accurately record water intake, water is often not recorded or reported in population surveys. In a systematic review of population surveys [25], over 50 % did not record water in children, 40 % in adolescents and 20 % in adults. This is perhaps a reflection of the difficulties in assessing the diet of children [32]; however, it does illustrate that until recently, water intake was not always considered important in population surveys. Undoubtedly with increasing interest in intake of all fluids including water, this will gradually change so that all fluids, including tap water, are recorded.

Data collection of intake and hydration status is an essential step in understanding the relationship between hydration and health. Biomarkers are increasingly being used in population surveys. For example, the Centre for Disease Control and the National Institute for Health are exploring the use of urine biomarkers to estimate population sodium intake [33]. Even though they are expensive and require ethical approval, biomarkers combined with intake data provide valuable information that is needed to set and evaluate public health policy [34]. A gold standard hydration marker has been described as elusive [35]; however, it has been suggested that a single, atypical plasma osmolality (Posm) threshold value of 301 mmol/kg would be a suitable indicator of dehydration [36]. The disadvantage of Posm is the need for a blood sample, which may be less acceptable to participants than urine sample collection. Therefore, research is being conducted to establish the feasibility of using urinary hydration markers feasible in population studies [37, 38]; these may include urine volume measurement or the number of bladder voids.

## Conclusions

With the increasing recognition of the importance of adequate water intake for health [1–3], it is vital that population surveys assess water and beverage and drink intake with reliable methods. While it is possible that existing methods may be capable of accurately capturing all such intake, this remains to be demonstrated by validating the methods for water and beverage and drink intake. Accurate assessment of water intake will facilitate research on water intake, hydration and its health impact. It will also provide factual arguments to perform a risk assessment related to ingredients present in certain drinks and beverages [39, 40]. An additional significance of having accurate fluid intake data is the fact that fluid intake data from population surveys are used by policy makers to set public health priorities and develop health improvement programs [41]; they are also used to monitor progress against targets and recommendations [42]. Currently, the recommendations for total water intake are adequate intakes. Ideally, more scientific evidence is generated in the future for the production of more defined recommendations, e.g., estimated average requirements, as opposed to adequate intakes.
